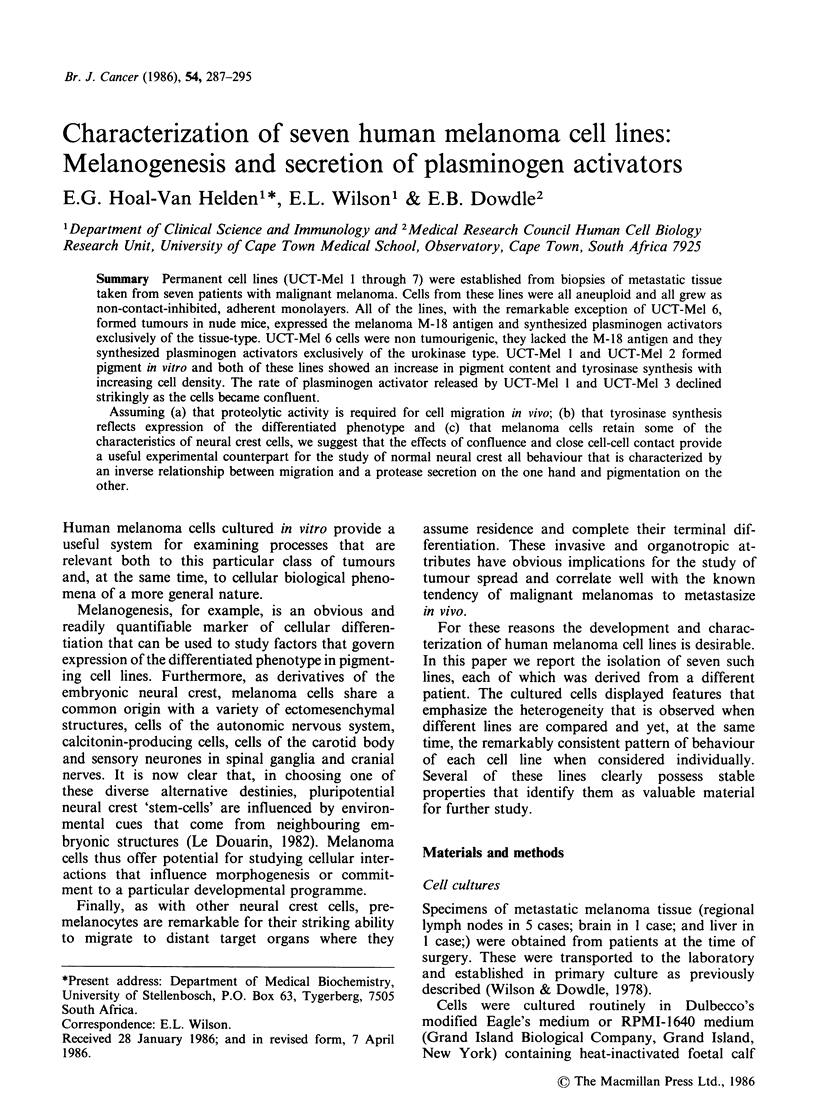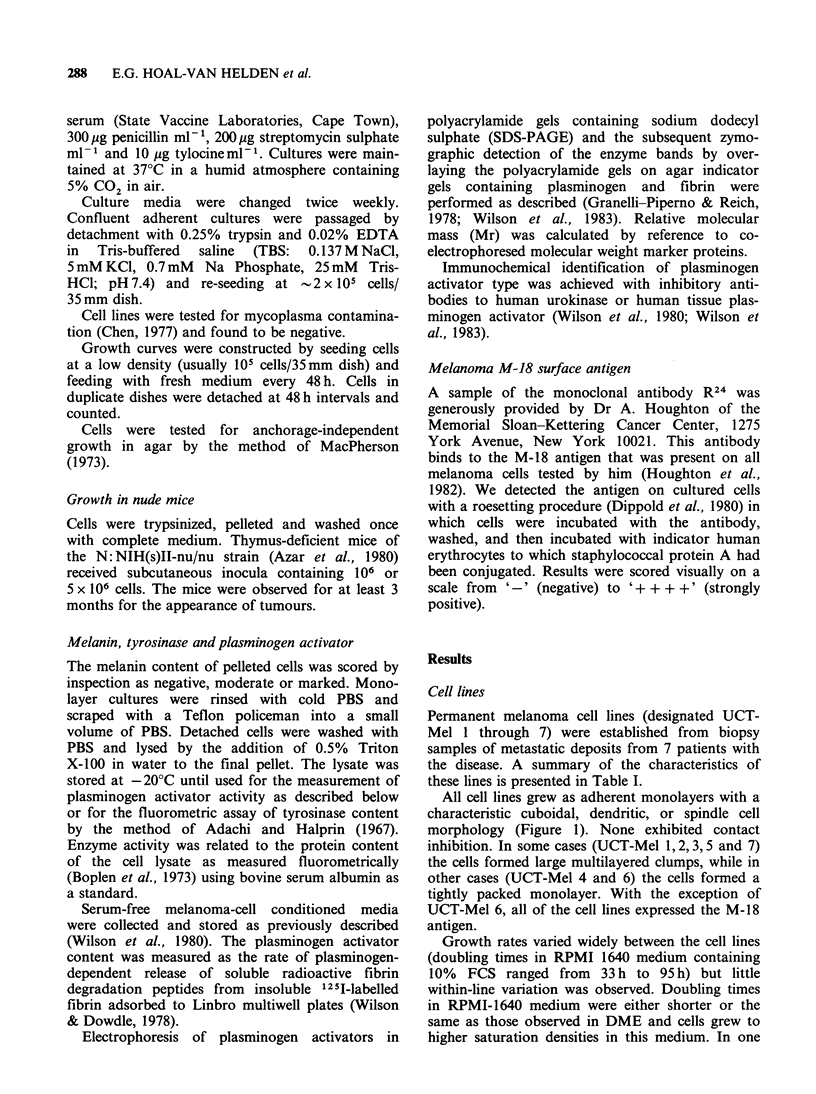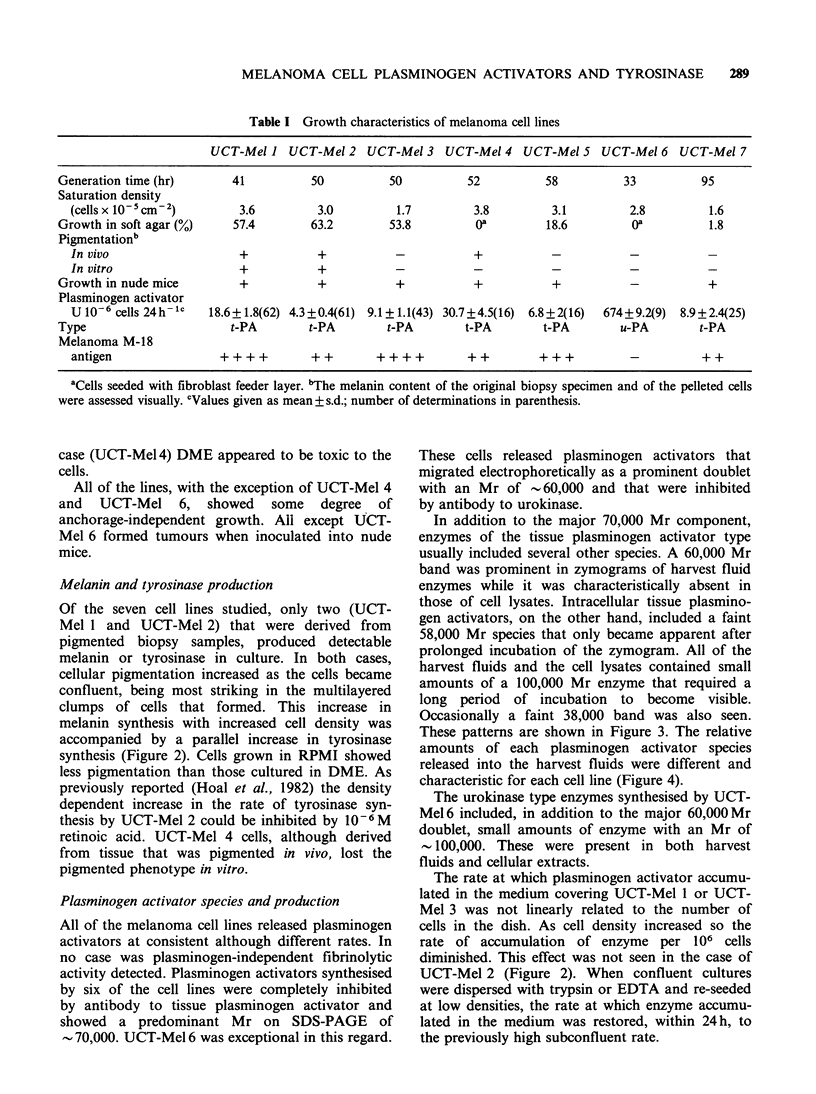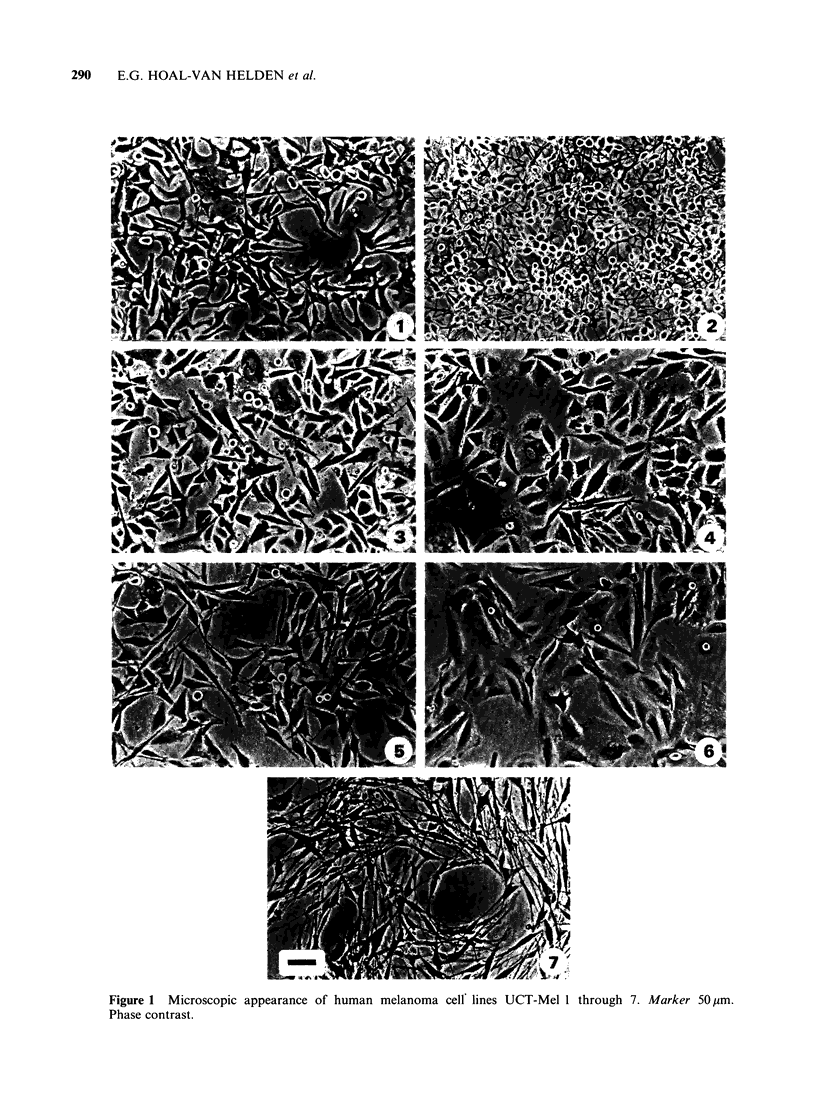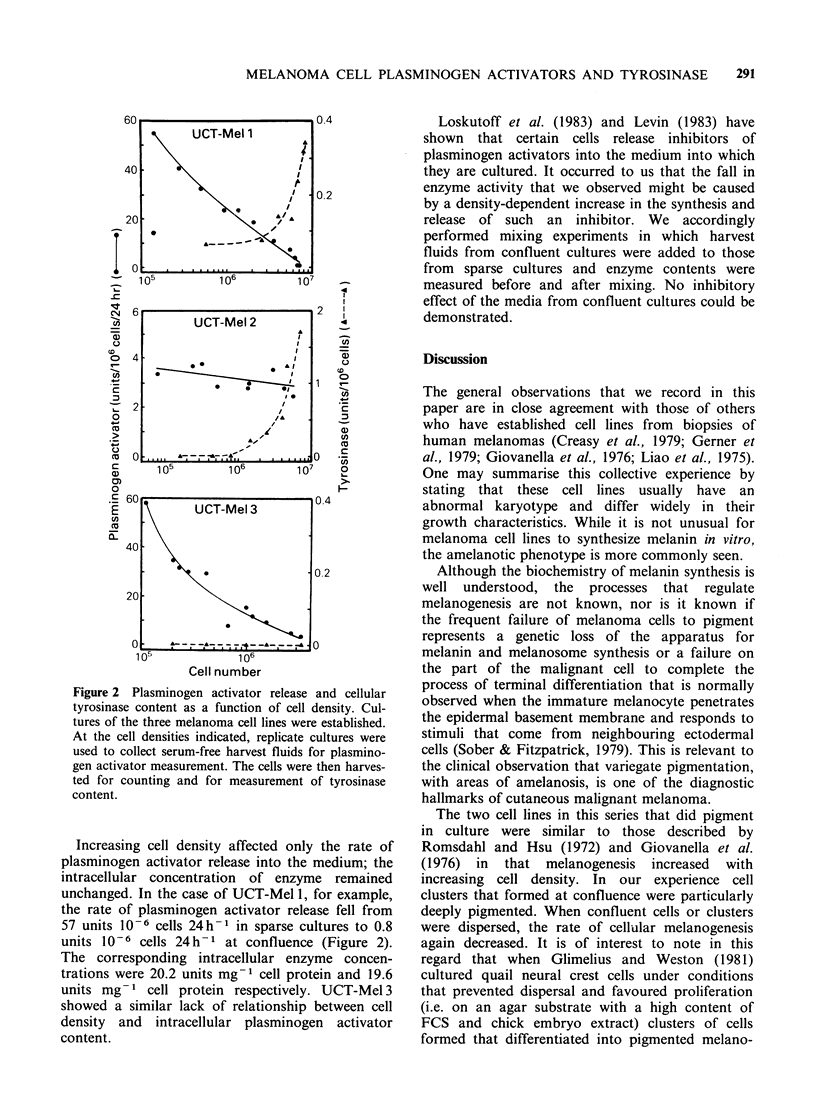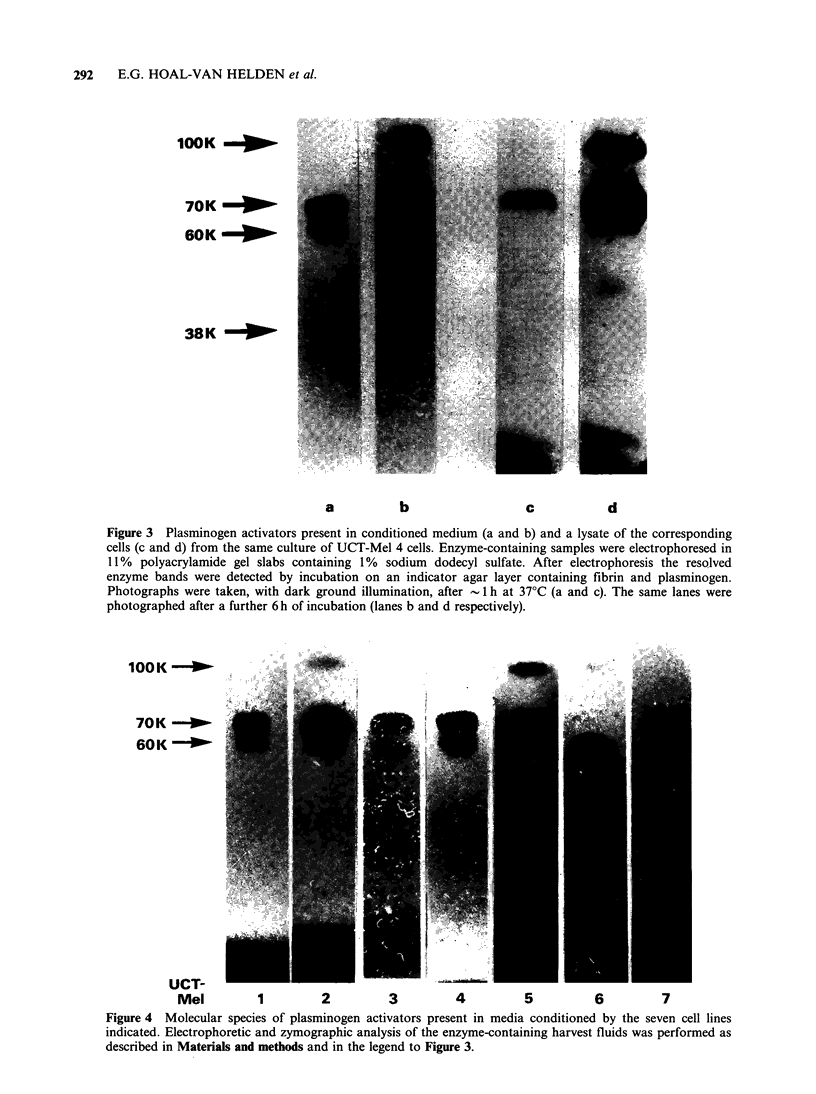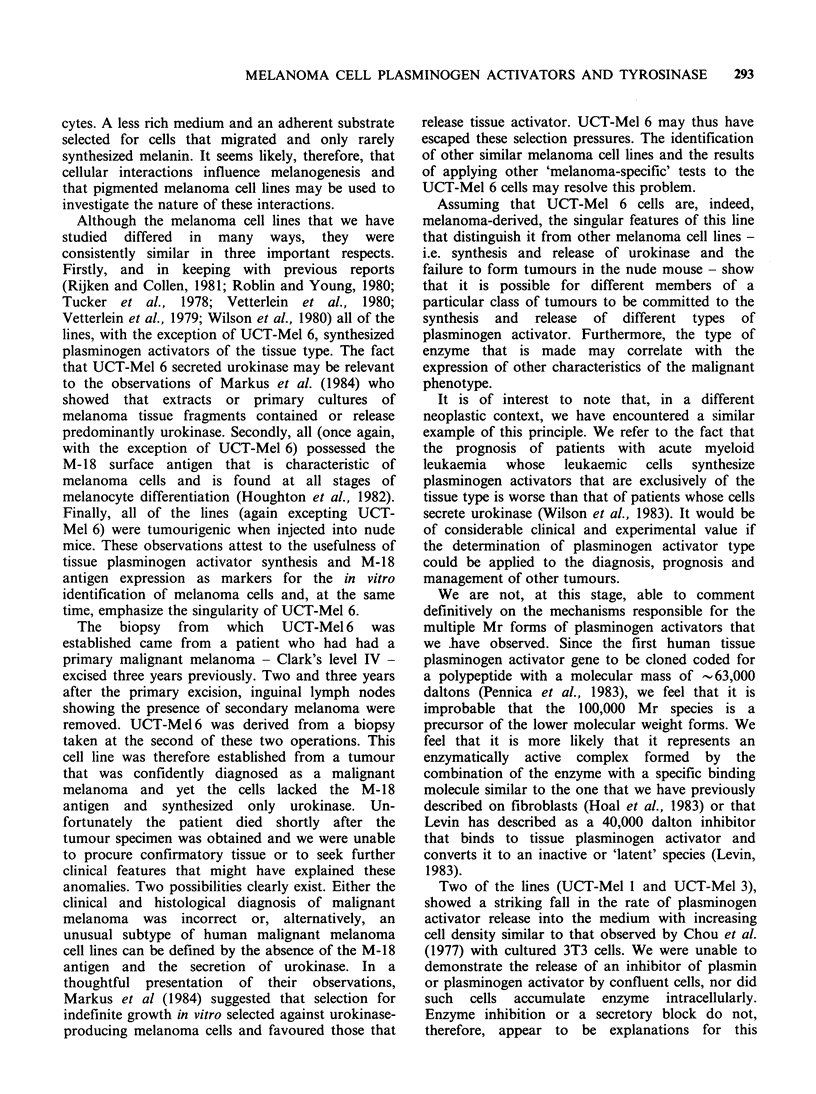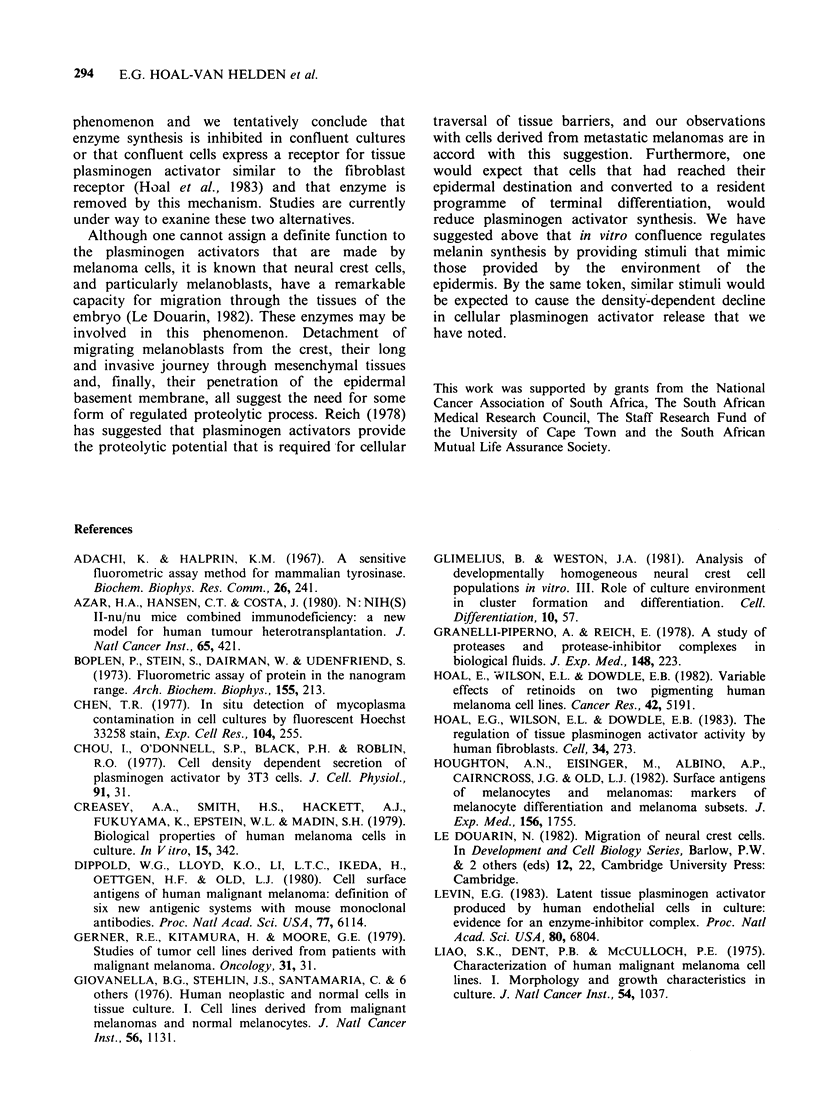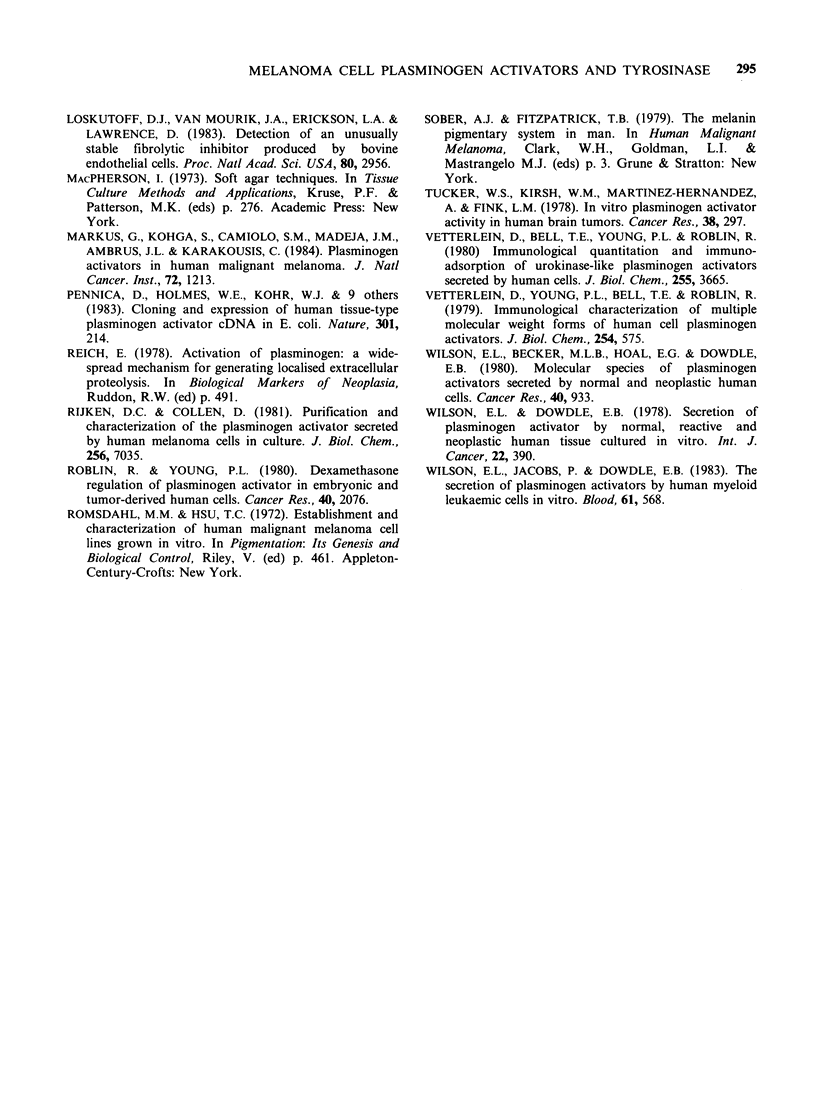# Characterization of seven human melanoma cell lines: melanogenesis and secretion of plasminogen activators.

**DOI:** 10.1038/bjc.1986.175

**Published:** 1986-08

**Authors:** E. G. Hoal-Van Helden, E. L. Wilson, E. B. Dowdle

## Abstract

**Images:**


Br. J. Cancer (1986), 54, 287–295

### Characterization of seven human melanoma cell lines: Melanogenesis and secretion of plasminogen activators

E.G. Hoal-Van Helden[1]*, E.L. Wilson[1] & E.B. Dowdle[2]

[1]*Department of Clinical Science and Immunology and* [2]*Medical Research Council Human Cell Biology Research Unit, University of Cape Town Medical School, Observatory, Cape Town, South Africa 7925*

**Summary** Permanent cell lines (UCT-Mel 1 through 7) were established from biopsies of metastatic tissue taken from seven patients with malignant melanoma. Cells from these lines were all aneuploid and all grew as non-contact-inhibited, adherent monolayers. All of the lines, with the remarkable exception of UCT-Mel 6, formed tumours in nude mice, expressed the melanoma M-18 antigen and synthesized plasminogen activators exclusively of the tissue-type. UCT-Mel 6 cells were non tumourigenic, they lacked the M-18 antigen and they synthesized plasminogen activators exclusively of the urokinase type. UCT-Mel 1 and UCT-Mel 2 formed pigment *in vitro* and both of these lines showed an increase in pigment content and tyrosinase synthesis with increasing cell density. The rate of plasminogen activator released by UCT-Mel 1 and UCT-Mel 3 declined strikingly as the cells became confluent.

Assuming (a) that proteolytic activity is required for cell migration *in vivo*; (b) that tyrosinase synthesis reflects expression of the differentiated phenotype and (c) that melanoma cells retain some of the characteristics of neural crest cells, we suggest that the effects of confluence and close cell-cell contact provide a useful experimental counterpart for the study of normal neural crest all behaviour that is characterized by an inverse relationship between migration and a protease secretion on the one hand and pigmentation on the other.

Human melanoma cells cultured *in vitro* provide a useful system for examining processes that are relevant both to this particular class of tumours and, at the same time, to cellular biological phenomena of a more general nature.

Melanogenesis, for example, is an obvious and readily quantifiable marker of cellular differentiation that can be used to study factors that govern expression of the differentiated phenotype in pigmenting cell lines. Furthermore, as derivatives of the embryonic neural crest, melanoma cells share a common origin with a variety of ectomesenchymal structures, cells of the autonomic nervous system, calcitonin-producing cells, cells of the carotid body and sensory neurones in spinal ganglia and cranial nerves. It is now clear that, in choosing one of these diverse alternative destinies, pluripotential neural crest 'stem-cells' are influenced by environmental cues that come from neighbouring embryonic structures (Le Douarin, 1982). Melanoma cells thus offer potential for studying cellular interactions that influence morphogenesis or commitment to a particular developmental programme.

Finally, as with other neural crest cells, premelanocytes are remarkable for their striking ability to migrate to distant target organs where they assume residence and complete their terminal differentiation. These invasive and organotropic attributes have obvious implications for the study of tumour spread and correlate well with the known tendency of malignant melanomas to metastasize *in vivo*.

For these reasons the development and characterization of human melanoma cell lines is desirable. In this paper we report the isolation of seven such lines, each of which was derived from a different patient. The cultured cells displayed features that emphasize the heterogeneity that is observed when different lines are compared and yet, at the same time, the remarkably consistent pattern of behaviour of each cell line when considered individually. Several of these lines clearly possess stable properties that identify them as valuable material for further study.

### Materials and methods

### *Cell cultures*

Specimens of metastatic melanoma tissue (regional lymph nodes in 5 cases; brain in 1 case; and liver in 1 case;) were obtained from patients at the time of surgery. These were transported to the laboratory and established in primary culture as previously described (Wilson & Dowdle, 1978).

Cells were cultured routinely in Dulbecco's modified Eagle's medium or RPMI-1640 medium (Grand Island Biological Company, Grand Island, New York) containing heat-inactivated foetal calf

*Present address: Department of Medical Biochemistry, University of Stellenbosch, P.O. Box 63, Tygerberg, 7505 South Africa.

Correspondence: E.L. Wilson.

Received 28 January 1986; and in revised form, 7 April 1986.

© The Macmillan Press Ltd., 1986

serum (State Vaccine Laboratories, Cape Town), 300 μg penicillin $ml^{-1}$, 200 μg streptomycin sulphate $ml^{-1}$ and 10 μg tylocine $ml^{-1}$. Cultures were maintained at 37°C in a humid atmosphere containing 5% $CO_2$ in air.

Culture media were changed twice weekly. Confluent adherent cultures were passaged by detachment with 0.25% trypsin and 0.02% EDTA in Tris-buffered saline (TBS: 0.137 M NaCl, 5 mM KCl, 0.7 mM Na Phosphate, 25 mM Tris-HCl; pH 7.4) and re-seeding at $\sim 2 \times 10^5$ cells/35 mm dish.

Cell lines were tested for mycoplasma contamination (Chen, 1977) and found to be negative.

Growth curves were constructed by seeding cells at a low density (usually $10^5$ cells/35 mm dish) and feeding with fresh medium every 48 h. Cells in duplicate dishes were detached at 48 h intervals and counted.

Cells were tested for anchorage-independent growth in agar by the method of MacPherson (1973).

*Growth in nude mice*

Cells were trypsinized, pelleted and washed once with complete medium. Thymus-deficient mice of the N:NIH(s)II-nu/nu strain (Azar *et al.*, 1980) received subcutaneous inocula containing $10^6$ or $5 \times 10^6$ cells. The mice were observed for at least 3 months for the appearance of tumours.

*Melanin, tyrosinase and plasminogen activator*

The melanin content of pelleted cells was scored by inspection as negative, moderate or marked. Monolayer cultures were rinsed with cold PBS and scraped with a Teflon policeman into a small volume of PBS. Detached cells were washed with PBS and lysed by the addition of 0.5% Triton X-100 in water to the final pellet. The lysate was stored at −20°C until used for the measurement of plasminogen activator activity as described below or for the fluorometric assay of tyrosinase content by the method of Adachi and Halprin (1967). Enzyme activity was related to the protein content of the cell lysate as measured fluorometrically (Boplen *et al.*, 1973) using bovine serum albumin as a standard.

Serum-free melanoma-cell conditioned media were collected and stored as previously described (Wilson *et al.*, 1980). The plasminogen activator content was measured as the rate of plasminogen-dependent release of soluble radioactive fibrin degradation peptides from insoluble $^{125}$I-labelled fibrin adsorbed to Linbro multiwell plates (Wilson & Dowdle, 1978).

Electrophoresis of plasminogen activators in polyacrylamide gels containing sodium dodecyl sulphate (SDS-PAGE) and the subsequent zymographic detection of the enzyme bands by overlaying the polyacrylamide gels on agar indicator gels containing plasminogen and fibrin were performed as described (Granelli–Piperno & Reich, 1978; Wilson *et al.*, 1983). Relative molecular mass (Mr) was calculated by reference to co-electrophoresed molecular weight marker proteins.

Immunochemical identification of plasminogen activator type was achieved with inhibitory antibodies to human urokinase or human tissue plasminogen activator (Wilson *et al.*, 1980; Wilson *et al.*, 1983).

*Melanoma M-18 surface antigen*

A sample of the monoclonal antibody $R^{24}$ was generously provided by Dr A. Houghton of the Memorial Sloan–Kettering Cancer Center, 1275 York Avenue, New York 10021. This antibody binds to the M-18 antigen that was present on all melanoma cells tested by him (Houghton *et al.*, 1982). We detected the antigen on cultured cells with a roesetting procedure (Dippold *et al.*, 1980) in which cells were incubated with the antibody, washed, and then incubated with indicator human erythrocytes to which staphylococcal protein A had been conjugated. Results were scored visually on a scale from '−' (negative) to '++++' (strongly positive).

### Results

*Cell lines*

Permanent melanoma cell lines (designated UCT-Mel 1 through 7) were established from biopsy samples of metastatic deposits from 7 patients with the disease. A summary of the characteristics of these lines is presented in Table I.

All cell lines grew as adherent monolayers with a characteristic cuboidal, dendritic, or spindle cell morphology (Figure 1). None exhibited contact inhibition. In some cases (UCT-Mel 1, 2, 3, 5 and 7) the cells formed large multilayered clumps, while in other cases (UCT-Mel 4 and 6) the cells formed a tightly packed monolayer. With the exception of UCT-Mel 6, all of the cell lines expressed the M-18 antigen.

Growth rates varied widely between the cell lines (doubling times in RPMI 1640 medium containing 10% FCS ranged from 33 h to 95 h) but little within-line variation was observed. Doubling times in RPMI-1640 medium were either shorter or the same as those observed in DME and cells grew to higher saturation densities in this medium. In one

**Table I** Growth characteristics of melanoma cell lines

| | *UCT-Mel 1* | *UCT-Mel 2* | *UCT-Mel 3* | *UCT-Mel 4* | *UCT-Mel 5* | *UCT-Mel 6* | *UCT-Mel 7* |
|---|---|---|---|---|---|---|---|
| Generation time (hr) | 41 | 50 | 50 | 52 | 58 | 33 | 95 |
| Saturation density | | | | | | | |
| (cells × $10^{-5}$ $cm^{-2}$) | 3.6 | 3.0 | 1.7 | 3.8 | 3.1 | 2.8 | 1.6 |
| Growth in soft agar (%) | 57.4 | 63.2 | 53.8 | 0[a] | 18.6 | 0[a] | 1.8 |
| Pigmentation[b] | | | | | | | |
| *In vivo* | + | + | – | + | – | – | – |
| *In vitro* | + | + | – | – | – | – | – |
| Growth in nude mice | + | + | + | + | + | – | + |
| Plasminogen activator | | | | | | | |
| U $10^{-6}$ cells 24 $h^{-1}$[c] | 18.6 ± 1.8(62) | 4.3 ± 0.4(61) | 9.1 ± 1.1(43) | 30.7 ± 4.5(16) | 6.8 ± 2(16) | 674 ± 9.2(9) | 8.9 ± 2.4(25) |
| Type | *t*-PA | *t*-PA | *t*-PA | t-PA | t-PA | *u*-PA | *t*-PA |
| Melanoma M-18 | | | | | | | |
| antigen | ++++ | ++ | ++++ | ++ | +++ | – | ++ |

[a]Cells seeded with fibroblast feeder layer. [b]The melanin content of the original biopsy specimen and of the pelleted cells were assessed visually. [c]Values given as mean ± s.d.; number of determinations in parenthesis.

case (UCT-Mel 4) DME appeared to be toxic to the cells.

All of the lines, with the exception of UCT-Mel 4 and UCT-Mel 6, showed some degree of anchorage-independent growth. All except UCT-Mel 6 formed tumours when inoculated into nude mice.

### *Melanin and tyrosinase production*

Of the seven cell lines studied, only two (UCT-Mel 1 and UCT-Mel 2) that were derived from pigmented biopsy samples, produced detectable melanin or tyrosinase in culture. In both cases, cellular pigmentation increased as the cells became confluent, being most striking in the multilayered clumps of cells that formed. This increase in melanin synthesis with increased cell density was accompanied by a parallel increase in tyrosinase synthesis (Figure 2). Cells grown in RPMI showed less pigmentation than those cultured in DME. As previously reported (Hoal *et al.*, 1982) the density dependent increase in the rate of tyrosinase synthesis by UCT-Mel 2 could be inhibited by $10^{-6}$ M retinoic acid. UCT-Mel 4 cells, although derived from tissue that was pigmented *in vivo,* lost the pigmented phenotype *in vitro.*

### *Plasminogen activator species and production*

All of the melanoma cell lines released plasminogen activators at consistent although different rates. In no case was plasminogen-independent fibrinolytic activity detected. Plasminogen activators synthesised by six of the cell lines were completely inhibited by antibody to tissue plasminogen activator and showed a predominant Mr on SDS-PAGE of ~70,000. UCT-Mel 6 was exceptional in this regard. These cells released plasminogen activators that migrated electrophoretically as a prominent doublet with an Mr of ~60,000 and that were inhibited by antibody to urokinase.

In addition to the major 70,000 Mr component, enzymes of the tissue plasminogen activator type usually included several other species. A 60,000 Mr band was prominent in zymograms of harvest fluid enzymes while it was characteristically absent in those of cell lysates. Intracellular tissue plasminogen activators, on the other hand, included a faint 58,000 Mr species that only became apparent after prolonged incubation of the zymogram. All of the harvest fluids and the cell lysates contained small amounts of a 100,000 Mr enzyme that required a long period of incubation to become visible. Occasionally a faint 38,000 band was also seen. These patterns are shown in Figure 3. The relative amounts of each plasminogen activator species released into the harvest fluids were different and characteristic for each cell line (Figure 4).

The urokinase type enzymes synthesised by UCT-Mel 6 included, in addition to the major 60,000 Mr doublet, small amounts of enzyme with an Mr of ~100,000. These were present in both harvest fluids and cellular extracts.

The rate at which plasminogen activator accumulated in the medium covering UCT-Mel 1 or UCT-Mel 3 was not linearly related to the number of cells in the dish. As cell density increased so the rate of accumulation of enzyme per $10^6$ cells diminished. This effect was not seen in the case of UCT-Mel 2 (Figure 2). When confluent cultures were dispersed with trypsin or EDTA and re-seeded at low densities, the rate at which enzyme accumulated in the medium was restored, within 24 h, to the previously high subconfluent rate.

**Figure 1** Microscopic appearance of human melanoma cell lines UCT-Mel 1 through 7. *Marker* 50 $\mu$m. Phase contrast.

**Figure 2** Plasminogen activator release and cellular tyrosinase content as a function of cell density. Cultures of the three melanoma cell lines were established. At the cell densities indicated, replicate cultures were used to collect serum-free harvest fluids for plasminogen activator measurement. The cells were then harvested for counting and for measurement of tyrosinase content.

Increasing cell density affected only the rate of plasminogen activator release into the medium; the intracellular concentration of enzyme remained unchanged. In the case of UCT-Mel 1, for example, the rate of plasminogen activator release fell from 57 units $10^{-6}$ cells $24\,h^{-1}$ in sparse cultures to 0.8 units $10^{-6}$ cells $24\,h^{-1}$ at confluence (Figure 2). The corresponding intracellular enzyme concentrations were 20.2 units $mg^{-1}$ cell protein and 19.6 units $mg^{-1}$ cell protein respectively. UCT-Mel 3 showed a similar lack of relationship between cell density and intracellular plasminogen activator content.

Loskutoff *et al.* (1983) and Levin (1983) have shown that certain cells release inhibitors of plasminogen activators into the medium into which they are cultured. It occurred to us that the fall in enzyme activity that we observed might be caused by a density-dependent increase in the synthesis and release of such an inhibitor. We accordingly performed mixing experiments in which harvest fluids from confluent cultures were added to those from sparse cultures and enzyme contents were measured before and after mixing. No inhibitory effect of the media from confluent cultures could be demonstrated.

### Discussion

The general observations that we record in this paper are in close agreement with those of others who have established cell lines from biopsies of human melanomas (Creasy *et al.*, 1979; Gerner *et al.*, 1979; Giovanella *et al.*, 1976; Liao *et al.*, 1975). One may summarise this collective experience by stating that these cell lines usually have an abnormal karyotype and differ widely in their growth characteristics. While it is not unusual for melanoma cell lines to synthesize melanin *in vitro*, the amelanotic phenotype is more commonly seen.

Although the biochemistry of melanin synthesis is well understood, the processes that regulate melanogenesis are not known, nor is it known if the frequent failure of melanoma cells to pigment represents a genetic loss of the apparatus for melanin and melanosome synthesis or a failure on the part of the malignant cell to complete the process of terminal differentiation that is normally observed when the immature melanocyte penetrates the epidermal basement membrane and responds to stimuli that come from neighbouring ectodermal cells (Sober & Fitzpatrick, 1979). This is relevant to the clinical observation that variegate pigmentation, with areas of amelanosis, is one of the diagnostic hallmarks of cutaneous malignant melanoma.

The two cell lines in this series that did pigment in culture were similar to those described by Romsdahl and Hsu (1972) and Giovanella *et al.* (1976) in that melanogenesis increased with increasing cell density. In our experience cell clusters that formed at confluence were particularly deeply pigmented. When confluent cells or clusters were dispersed, the rate of cellular melanogenesis again decreased. It is of interest to note in this regard that when Glimelius and Weston (1981) cultured quail neural crest cells under conditions that prevented dispersal and favoured proliferation (i.e. on an agar substrate with a high content of FCS and chick embryo extract) clusters of cells formed that differentiated into pigmented melano-

**Figure 3** Plasminogen activators present in conditioned medium (a and b) and a lysate of the corresponding cells (c and d) from the same culture of UCT-Mel 4 cells. Enzyme-containing samples were electrophoresed in 11% polyacrylamide gel slabs containing 1% sodium dodecyl sulfate. After electrophoresis the resolved enzyme bands were detected by incubation on an indicator agar layer containing fibrin and plasminogen. Photographs were taken, with dark ground illumination, after ~1 h at 37°C (a and c). The same lanes were photographed after a further 6 h of incubation (lanes b and d respectively).

**Figure 4** Molecular species of plasminogen activators present in media conditioned by the seven cell lines indicated. Electrophoretic and zymographic analysis of the enzyme-containing harvest fluids was performed as described in **Materials and methods** and in the legend to **Figure 3**.

cytes. A less rich medium and an adherent substrate selected for cells that migrated and only rarely synthesized melanin. It seems likely, therefore, that cellular interactions influence melanogenesis and that pigmented melanoma cell lines may be used to investigate the nature of these interactions.

Although the melanoma cell lines that we have studied differed in many ways, they were consistently similar in three important respects. Firstly, and in keeping with previous reports (Rijken and Collen, 1981; Roblin and Young, 1980; Tucker *et al.*, 1978; Vetterlein *et al.*, 1980; Vetterlein *et al.*, 1979; Wilson *et al.*, 1980) all of the lines, with the exception of UCT-Mel 6, synthesized plasminogen activators of the tissue type. The fact that UCT-Mel 6 secreted urokinase may be relevant to the observations of Markus *et al.* (1984) who showed that extracts or primary cultures of melanoma tissue fragments contained or release predominantly urokinase. Secondly, all (once again, with the exception of UCT-Mel 6) possessed the M-18 surface antigen that is characteristic of melanoma cells and is found at all stages of melanocyte differentiation (Houghton *et al.*, 1982). Finally, all of the lines (again excepting UCT-Mel 6) were tumourigenic when injected into nude mice. These observations attest to the usefulness of tissue plasminogen activator synthesis and M-18 antigen expression as markers for the *in vitro* identification of melanoma cells and, at the same time, emphasize the singularity of UCT-Mel 6.

The biopsy from which UCT-Mel 6 was established came from a patient who had had a primary malignant melanoma – Clark's level IV – excised three years previously. Two and three years after the primary excision, inguinal lymph nodes showing the presence of secondary melanoma were removed. UCT-Mel 6 was derived from a biopsy taken at the second of these two operations. This cell line was therefore established from a tumour that was confidently diagnosed as a malignant melanoma and yet the cells lacked the M-18 antigen and synthesized only urokinase. Unfortunately the patient died shortly after the tumour specimen was obtained and we were unable to procure confirmatory tissue or to seek further clinical features that might have explained these anomalies. Two possibilities clearly exist. Either the clinical and histological diagnosis of malignant melanoma was incorrect or, alternatively, an unusual subtype of human malignant melanoma cell lines can be defined by the absence of the M-18 antigen and the secretion of urokinase. In a thoughtful presentation of their observations, Markus *et al* (1984) suggested that selection for indefinite growth *in vitro* selected against urokinase-producing melanoma cells and favoured those that release tissue activator. UCT-Mel 6 may thus have escaped these selection pressures. The identification of other similar melanoma cell lines and the results of applying other 'melanoma-specific' tests to the UCT-Mel 6 cells may resolve this problem.

Assuming that UCT-Mel 6 cells are, indeed, melanoma-derived, the singular features of this line that distinguish it from other melanoma cell lines – i.e. synthesis and release of urokinase and the failure to form tumours in the nude mouse – show that it is possible for different members of a particular class of tumours to be committed to the synthesis and release of different types of plasminogen activator. Furthermore, the type of enzyme that is made may correlate with the expression of other characteristics of the malignant phenotype.

It is of interest to note that, in a different neoplastic context, we have encountered a similar example of this principle. We refer to the fact that the prognosis of patients with acute myeloid leukaemia whose leukaemic cells synthesize plasminogen activators that are exclusively of the tissue type is worse than that of patients whose cells secrete urokinase (Wilson *et al.*, 1983). It would be of considerable clinical and experimental value if the determination of plasminogen activator type could be applied to the diagnosis, prognosis and management of other tumours.

We are not, at this stage, able to comment definitively on the mechanisms responsible for the multiple Mr forms of plasminogen activators that we have observed. Since the first human tissue plasminogen activator gene to be cloned coded for a polypeptide with a molecular mass of ~63,000 daltons (Pennica *et al.*, 1983), we feel that it is improbable that the 100,000 Mr species is a precursor of the lower molecular weight forms. We feel that it is more likely that it represents an enzymatically active complex formed by the combination of the enzyme with a specific binding molecule similar to the one that we have previously described on fibroblasts (Hoal *et al.*, 1983) or that Levin has described as a 40,000 dalton inhibitor that binds to tissue plasminogen activator and converts it to an inactive or 'latent' species (Levin, 1983).

Two of the lines (UCT-Mel 1 and UCT-Mel 3), showed a striking fall in the rate of plasminogen activator release into the medium with increasing cell density similar to that observed by Chou *et al.* (1977) with cultured 3T3 cells. We were unable to demonstrate the release of an inhibitor of plasmin or plasminogen activator by confluent cells, nor did such cells accumulate enzyme intracellularly. Enzyme inhibition or a secretory block do not, therefore, appear to be explanations for this phenomenon and we tentatively conclude that enzyme synthesis is inhibited in confluent cultures or that confluent cells express a receptor for tissue plasminogen activator similar to the fibroblast receptor (Hoal *et al.*, 1983) and that enzyme is removed by this mechanism. Studies are currently under way to exanine these two alternatives.

Although one cannot assign a definite function to the plasminogen activators that are made by melanoma cells, it is known that neural crest cells, and particularly melanoblasts, have a remarkable capacity for migration through the tissues of the embryo (Le Douarin, 1982). These enzymes may be involved in this phenomenon. Detachment of migrating melanoblasts from the crest, their long and invasive journey through mesenchymal tissues and, finally, their penetration of the epidermal basement membrane, all suggest the need for some form of regulated proteolytic process. Reich (1978) has suggested that plasminogen activators provide the proteolytic potential that is required for cellular traversal of tissue barriers, and our observations with cells derived from metastatic melanomas are in accord with this suggestion. Furthermore, one would expect that cells that had reached their epidermal destination and converted to a resident programme of terminal differentiation, would reduce plasminogen activator synthesis. We have suggested above that *in vitro* confluence regulates melanin synthesis by providing stimuli that mimic those provided by the environment of the epidermis. By the same token, similar stimuli would be expected to cause the density-dependent decline in cellular plasminogen activator release that we have noted.

This work was supported by grants from the National Cancer Association of South Africa, The South African Medical Research Council, The Staff Research Fund of the University of Cape Town and the South African Mutual Life Assurance Society.

### References

ADACHI, K. & HALPRIN, K.M. (1967). A sensitive fluorometric assay method for mammalian tyrosinase. *Biochem. Biophys. Res. Comm.*, **26**, 241.

AZAR, H.A., HANSEN, C.T. & COSTA, J. (1980). N:NIH(S) II-nu/nu mice combined immunodeficiency: a new model for human tumour heterotransplantation. *J. Natl Cancer Inst.*, **65**, 421.

BOPLEN, P., STEIN, S., DAIRMAN, W. & UDENFRIEND, S. (1973). Fluorometric assay of protein in the nanogram range. *Arch. Biochem. Biophys.*, **155**, 213.

CHEN, T.R. (1977). In situ detection of mycoplasma contamination in cell cultures by fluorescent Hoechst 33258 stain, *Exp. Cell Res.*, **104**, 255.

CHOU, I., O'DONNELL, S.P., BLACK, P.H. & ROBLIN, R.O. (1977). Cell density dependent secretion of plasminogen activator by 3T3 cells. *J. Cell. Physiol.*, **91**, 31.

CREASEY, A.A., SMITH, H.S., HACKETT, A.J., FUKUYAMA, K., EPSTEIN, W.L. & MADIN, S.H. (1979). Biological properties of human melanoma cells in culture. *In Vitro*, **15**, 342.

DIPPOLD, W.G., LLOYD, K.O., LI, L.T.C., IKEDA, H., OETTGEN, H.F. & OLD, L.J. (1980). Cell surface antigens of human malignant melanoma: definition of six new antigenic systems with mouse monoclonal antibodies. *Proc. Natl Acad. Sci. USA*, **77**, 6114.

GERNER, R.E., KITAMURA, H. & MOORE, G.E. (1979). Studies of tumor cell lines derived from patients with malignant melanoma. *Oncology*, **31**, 31.

GIOVANELLA, B.G., STEHLIN, J.S., SANTAMARIA, C. & 6 others (1976). Human neoplastic and normal cells in tissue culture. I. Cell lines derived from malignant melanomas and normal melanocytes. *J. Natl Cancer Inst.*, **56**, 1131.

GLIMELIUS, B. & WESTON, J.A. (1981). Analysis of developmentally homogeneous neural crest cell populations *in vitro*. III. Role of culture environment in cluster formation and differentiation. *Cell. Differentiation*, **10**, 57.

GRANELLI-PIPERNO, A. & REICH, E. (1978). A study of proteases and protease-inhibitor complexes in biological fluids. *J. Exp. Med.*, **148**, 223.

HOAL, E., WILSON, E.L. & DOWDLE, E.B. (1982). Variable effects of retinoids on two pigmenting human melanoma cell lines. *Cancer Res.*, **42**, 5191.

HOAL, E.G., WILSON, E.L. & DOWDLE, E.B. (1983). The regulation of tissue plasminogen activator activity by human fibroblasts. *Cell*, **34**, 273.

HOUGHTON, A.N., EISINGER, M., ALBINO, A.P., CAIRNCROSS, J.G. & OLD, L.J. (1982). Surface antigens of melanocytes and melanomas: markers of melanocyte differentiation and melanoma subsets. *J. Exp. Med.*, **156**, 1755.

LE DOUARIN, N. (1982). Migration of neural crest cells. In *Development and Cell Biology Series*, Barlow, P.W. & 2 others (eds) **12**, 22, Cambridge University Press: Cambridge.

LEVIN, E.G. (1983). Latent tissue plasminogen activator produced by human endothelial cells in culture: evidence for an enzyme-inhibitor complex. *Proc. Natl Acad. Sci. USA*, **80**, 6804.

LIAO, S.K., DENT, P.B. & McCULLOCH, P.E. (1975). Characterization of human malignant melanoma cell lines. I. Morphology and growth characteristics in culture. *J. Natl Cancer Inst.*, **54**, 1037.

LOSKUTOFF, D.J., VAN MOURIK, J.A., ERICKSON, L.A. & LAWRENCE, D. (1983). Detection of an unusually stable fibrolytic inhibitor produced by bovine endothelial cells. *Proc. Natl Acad. Sci. USA*, **80**, 2956.

MacPHERSON, I. (1973). Soft agar techniques. In *Tissue Culture Methods and Applications*, Kruse, P.F. & Patterson, M.K. (eds) p. 276. Academic Press: New York.

MARKUS, G., KOHGA, S., CAMIOLO, S.M., MADEJA, J.M., AMBRUS, J.L. & KARAKOUSIS, C. (1984). Plasminogen activators in human malignant melanoma. *J. Natl Cancer. Inst.*, **72**, 1213.

PENNICA, D., HOLMES, W.E., KOHR, W.J. & 9 others (1983). Cloning and expression of human tissue-type plasminogen activator cDNA in E. coli. *Nature*, **301**, 214.

REICH, E. (1978). Activation of plasminogen: a widespread mechanism for generating localised extracellular proteolysis. In *Biological Markers of Neoplasia*, Ruddon, R.W. (ed) p. 491.

RIJKEN, D.C. & COLLEN, D. (1981). Purification and characterization of the plasminogen activator secreted by human melanoma cells in culture. *J. Biol. Chem.*, **256**, 7035.

ROBLIN, R. & YOUNG, P.L. (1980). Dexamethasone regulation of plasminogen activator in embryonic and tumor-derived human cells. *Cancer Res.*, **40**, 2076.

ROMSDAHL, M.M. & HSU, T.C. (1972). Establishment and characterization of human malignant melanoma cell lines grown in vitro. In *Pigmentation: Its Genesis and Biological Control*, Riley, V. (ed) p. 461. Appleton-Century-Crofts: New York.

SOBER, A.J. & FITZPATRICK, T.B. (1979). The melanin pigmentary system in man. In *Human Malignant Melanoma*, Clark, W.H., Goldman, L.I. & Mastrangelo M.J. (eds) p. 3. Grune & Stratton: New York.

TUCKER, W.S., KIRSH, W.M., MARTINEZ-HERNANDEZ, A. & FINK, L.M. (1978). In vitro plasminogen activator activity in human brain tumors. *Cancer Res.*, **38**, 297.

VETTERLEIN, D., BELL, T.E., YOUNG, P.L. & ROBLIN, R. (1980) Immunological quantitation and immunoadsorption of urokinase-like plasminogen activators secreted by human cells. *J. Biol. Chem.*, **255**, 3665.

VETTERLEIN, D., YOUNG, P.L., BELL, T.E. & ROBLIN, R. (1979). Immunological characterization of multiple molecular weight forms of human cell plasminogen activators. *J. Biol. Chem.*, **254**, 575.

WILSON, E.L., BECKER, M.L.B., HOAL, E.G. & DOWDLE, E.B. (1980). Molecular species of plasminogen activators secreted by normal and neoplastic human cells. *Cancer Res.*, **40**, 933.

WILSON, E.L. & DOWDLE, E.B. (1978). Secretion of plasminogen activator by normal, reactive and neoplastic human tissue cultured in vitro. *Int. J. Cancer*, **22**, 390.

WILSON, E.L., JACOBS, P. & DOWDLE, E.B. (1983). The secretion of plasminogen activators by human myeloid leukaemic cells in vitro. *Blood*, **61**, 568.